# Supplementary material for: Pseudomonas aeruginosa Resistance to Bacteriophages and Its Prevention by Strategic Therapeutic Cocktail Formulation
Source: Antibiotics (Basel). 2021 Feb 2;10(2):145. doi: 10.3390/antibiotics10020145 (PMC7912912; doi:10.3390/antibiotics10020145)
Supplement: Supplementary file 1 [file antibiotics-10-00145-s001.pdf]

antibiotics

Table S1. *P. aeruginosa* Receptor Mutations Conferring Phage-Resistance.

Phage Treatment	Mutation Type	Gene/s	Receptor	Role of Gene	Cost to Fitness	Bacterial Genetic Background	Reference
Sequential 2 phages	80bp deletion	*galU*	LPS	LPS core biosynthesis	N/A	PAO1	[84]
Sequential 2 phages	Nonsense	*galU*	LPS	LPS core biosynthesis	N/A	PAO1	[84]
Single Phage	Frameshift	*Ssg*	LPS	OSA biosynthesis	N/A	PAO1	[84]
Sequential 2 phages	91bp deletion	*wapH*	LPS	Probable outer core biosynthesis	N/A	PAO1	[84]
Sequential 2 phages	Missense	*rmlA*	LPS	LPS core biosynthesis	N/A	PAO1	[84]
2 phage cocktail	Frameshift	*wbpL*	LPS	LPS CPA and OSA biosynthesis	N/A	PAO1	[84]
Sequential 2 phages	Nonsense	*wzy*	LPS	LPS OSA biosynthesis	N/A	PAO1	[84]
2 phage cocktail	Frameshift	*wzy*	LPS	LPS OSA biosynthesis	N/A	PAO1	[84]
Sequential 2 phages	Missense	*pilB*	T4P	Pilus extension	N/A	PAO1	[84]
Sequential 2 phages	12bp insertion	*pilE*	T4P	Minor pilin	N/A	PAO1	[84]
Sequential 2 phages	Missense	*pilN*	T4P	Pilus assembly	N/A	PAO1	[84]
Sequential 2 phages	Missense	*pilR*	T4P	Interacts with sensor to regulate the production of the pilus	N/A	PAO1	[84]
Sequential 2 phages	1,192bp deletion	*pilT and pilU*	T4P	Twitching motility	N/A	PAO1	[84]
Sequential 2 phages	Nonsense	*pilY1*	T4P	Pilus anti-retraction	N/A	PAO1	[84]
2 phage cocktail	Frameshift	*pilY1*	T4P	Pilus anti-retraction	N/A	PAO1	[84]
2 phage cocktail	250kbp deletion	*galU* and 200 other genes	LPS	N/A	N/A	PAO1	[84]
Single Phage	1bp Substitution	*wzy*	LPS	LPS OSA biosynthesis	Reduced biofilm formation	PA1	[85]
Single Phage	1bp insertion	*wbpL*	LPS	LPS CPA and OSA biosynthesis	N/A	PAO1	[83]
Single Phage	1bp insertion	*wzy*	LPS	LPS OSA biosynthesis	N/A	PAO1	[83]
Single Phage	4bp deletion	*pilB*	T4P	Motor protein powering pilus extension	N/A	PAO1	[83]
Single Phage	Nonsense	*pilB*	T4P	Motor protein powering pilus extension	N/A	PAO1	[83]
Single Phage	Missense	*pilT*	T4P	Motor protein powering pilus retraction	N/A	PAO1	[83]
Single Phage	1200bp deletion	*pilT*	T4P	Motor protein powering pilus retraction	N/A	PAO1	[83]
Single Phage	12bp deletion	*pilT*	T4P	Motor protein powering pilus retraction	N/A	PAO1	[83]

Single Phage	1bp deletion	*pilY1*	T4P	Pilus anti-retraction	N/A	PAO1	[83]
Single Phage	Missense	*pilJ*	T4P	Transduction of signals to system regulating type IV pilus motility	N/A	PAO1	[83]
Single Phage	Nonsense	*pilD*	T4P	Processing T4P prepilins	N/A	PAO1	[83]
Single Phage	183bp deletion	*pilQ*	T4P	Pilus outer membrane secretin pore	N/A	PAO1	[83]
Single Phage	35bp deletion	*fimV*	T4P	Involved in pilus assembly	N/A	PAO1	[83]
Single Phage	1bp deletion	*rpoN*	T4P	Regulates expression of major pilin protein	N/A	PAO1	[83]
Single Phage	Missense	*rpoN*	T4P	Regulates expression of major pilin protein	N/A	PAO1	[83]
Single Phage	1bp deletion	*pilR*	T4P	Interacts with sensor to regulate the production of the pilus	N/A	PAO1	[83]
Single Phage	Missense	*pilR*	T4P	Interacts with sensor to regulate the production of the pilus	N/A	PAO1	[83]
Single Phage	Nonsense	*pilS*	T4P	Sensor that interacts with regulator to produce the pilus	N/A	PAO1	[83]
Single Phage	Missense	PA1875	Putative Efflux Pump	N/A	N/A	PAO1	[83]
Single Phage	220kbp deletion	*galU*	LPS	LPS core biosynthesis	Less virulent in mouse model	PA1	[86]
13 Phage Cocktail	15bp deletion	*pilT*	T4P	Motor protein for pilus retraction	Twitching motility	CHA	[58]
13 Phage Cocktail	362kbp deletion	*galU*	LPS	LPS core biosynthesis	Decreased resistance to ciprofloxacin	CHA	[58]
3 Phage Cocktail	1bp insertion	Pseudogene	N/A	N/A	Reduced swimming, swarming and twitching motility	PAO1	[87]
	1bp substitution	*ldhA*	N/A	Cell metabolism			
	1bp substitution	*galU*	LPS	LPS core biosynthesis			
	1bp deletion	Hypothetical protein	N/A	N/A			
	3bp insertion	*mexT*	N/A	Transcriptional regulator			
	1bp substitution	Probable transcriptional regulator	N/A	N/A			
	1bp insertion	Hypothetical protein	N/A	N/A			
	1bp substitution	*pilE*	T4P	Type 4 fimbrial biogenesis			
3 Phage Cocktail	1bp insertion	Pseudogene	N/A	N/A	Reduced swimming, swarming and twitching motility	PAO1	[87]
	1bp substitution	*ldhA*	N/A	Cell metabolism			

	1bp substitution	*galU*	LPS	LPS core biosynthesis			
	1bp deletion	Hypothetical protein	N/A	N/A			
	3bp insertion	*mexT*	N/A	Transcriptional regulator			
	1bp substitution	Probable transcriptional regulator	N/A	N/A			
	1bp insertion	Hypothetical protein	N/A	N/A			
	1bp substitution	*pilO*	T4P	Type 4 fimbrial biogenesis			
3 Phage Cocktail	1bp insertion	Pseudogene	N/A	N/A	Reduced swimming, swarming and twitching motility	PAO1	[87]
	1bp substitution	*ldhA*	N/A	Cell metabolism			
	1bp substitution	*galU*	LPS	LPS core biosynthesis			
	1bp deletion	Hypothetical protein	N/A	N/A			
	3bp insertion	*mexT*	N/A	Transcriptional regulator			
	1bp substitution	Probable transcriptional regulator	N/A	N/A			
	1bp insertion	Hypothetical protein	N/A	N/A			
	1bp insertion	*pilC*	T4P	Type 4 fimbrial biogenesis			
3 Phage Cocktail	1bp insertion	Pseudogene	N/A	N/A	Reduced swimming, swarming and twitching motility	PAO1	[87]
	1bp substitution	*ldhA*	N/A	Cell metabolism			
	1bp substitution	*galU*	LPS	LPS core biosynthesis			
	1bp deletion	Hypothetical protein	N/A	N/A			
	3bp insertion	*mexT*	N/A	Transcriptional regulator			
	1bp substitution	Probable transcriptional regulator	N/A	N/A			
	1bp insertion	Hypothetical protein	N/A	N/A			
	1bp insertion	*pilM*	T4P	Type 4 fimbrial biogenesis			
5 Cocktail	1bp insertion	*wzy*	LPS	LPS OSA biosynthesis	N/A	PA1	[109]
	1bp substitution	*migA*	LPS	LPS core biosynthesis			
5 Cocktail	1bp substitution	*migA*	LPS	LPS core biosynthesis	N/A	PA1	[109]

	6bp insertion	*fimL*	Unknown	Unknown			
	1bp substitution	*wzy*	LPS	LPS OSA biosynthesis			
	3bp substitution	*gmd*	LPS	LPS CPA biosynthesis			
5 Cocktail	1bp substitution	*migA*	LPS	LPS core biosynthesis	N/A	PA1	[109]
	1bp substitution	*wzy*	LPS	LPS OSA biosynthesis			
	1bp substitution	*rmd*	LPS	LPS CPA biosynthesis			
5 Cocktail	1bp substitution	*migA*	LPS	LPS core biosynthesis	N/A	PA1	[109]
	1bp substitution	*wzy*	LPS	LPS OSA biosynthesis			
	1bp substitution	*PA5455*	Unknown	Unknown			
2 Phage Cocktail	1bp insertion	*wzy*	LPS	LPS OSA biosynthesis	N/A	PA01	[86]
2 Phage Cocktail	1bp insertion	*wzy*	LPS	LPS OSA biosynthesis	N/A	PA01	[86]
	1bp substitution	*migA*	LPS	LPS core biosynthesis	N/A	PA01	[86]
Single Phage	1bp insertion	*wzy*	LPS	LPS OSA biosynthesis	N/A	PA01	[86]
Single Phage	1bp insertion	*wzy*	LPS	LPS OSA biosynthesis	N/A	PA01	[86]
Single Phage	1bp insertion	*wbpL*	LPS	LPS CPA and OSA biosynthesis	N/A	PA01	[86]
Single Phage	1bp substitution	*wapH*	LPS	Probable outer core biosynthesis	N/A	PA01	[86]
2 Phage Cocktail	1bp substitution	*dnpA*	N/A	LPS core biosynthesis	N/A	PA01	[86]
4 Phage Cocktail	1bp insertion	*wzy*	LPS	LPS OSA biosynthesis	N/A	PA01	[86]
2 Phage Cocktail	1bp insertion	*mucA*	N/A	Anti-sigma factor; alginate regulation	N/A	PA01	[86]
2 Phage Cocktail	1bp insertion	*wzz2*	LPS	LPS OSA biosynthesis	N/A	PA01	[86]
2 Phage Cocktail	1bp insertion	*mucA*	N/A	Anti-sigma factor; alginate regulation	N/A	PA01	[86]
Single Phage	1bp substitution	*pgi*	N/A	Cell metabolism	N/A	PA01	[86]
Single Phage	1bp substitution	*mucA* intergenic *algU*	N/A	N/A	N/A	PA01	[86]
Single Phage	1bp substitution	*wzy*	LPS	LPS OSA biosynthesis	N/A	PA01	[86]
Single Phage	19bp deletion	*pilQ*	T4P	Outer membrane secretin pore within pilus structure	N/A	PA01	[86]
4 Phage Cocktail	1bp insertion	*wzy*	LPS	LPS OSA biosynthesis	N/A	PA01	[86]
	1bp substitution	*pilR*	T4P	Motility two-component response regulator			

2 Phage Cocktail	10bp deletion	*pilY1*	T4P	Pilus anti-retraction factor	N/A	PA01	[86]
	1bp substitution	*wzy*	LPS	LPS OSA biosynthesis			
4 Phage Cocktail	555bp deletion	*pilQ*	T4P	Pilus outer membrane secretin pore	N/A	PA01	[86]
4 Phage Cocktail	1bp deletion	*pilR*	T4P	Two-component response regulator	N/A	PA01	[86]
	213bp deletion	*algC*	LPS	O antigen and alginate biosynthesis			
2 Phage Cocktail	1bp insertion	*wzy*	LPS	LPS OSA biosynthesis	N/A	PA01	[86]
	1bp deletion	*wzy*	LPS	LPS OSA biosynthesis			
	109bp deletion	*pilY1*	T4P	Pilus anti-retraction factor			
2 Phage Cocktail	11bp insertion	*pilJ*	T4P	Motility two-component response signal transduction	N/A	PA01	[86]
	1bp insertion	*wzy*	LPS	LPS OSA biosynthesis			

Note: Where a row contains more than one mutation it is because a single escape mutant contained multiple mutations.
